# Christianson syndrome: A novel splicing variant of *SLC9A6* causes exon skipping in a Chinese boy and a literature review

**DOI:** 10.1002/jcla.24123

**Published:** 2021-11-17

**Authors:** Xiaoge Zhang, Xiaofang Wu, Hongli Liu, Tingting Song, Yongsheng Jiang, Hanhan He, Shaoqing Yang, Yun Xie

**Affiliations:** ^1^ Department of pediatrics Northwest Women’s and Children’s Hospital Xi’an China; ^2^ State Key Laboratory of Military Stomatology Department of Oral Biology School of Stomatology Clinic of Oral Rare and Genetic Diseases National Clinical Research Center for Oral Diseases The Fourth Military Medical University Xi'an China; ^3^ Department of clinical laboratory Northwest Women’s and Children’s Hospital Xi’an China

**Keywords:** chinese boy, christianson syndrome, novel splicing variant, *SLC9A6*

## Abstract

**Background:**

Variants in the endosomal solute carrier family 9 member A6 (*SLC9A6*)/(Na^+^,K^+^)/H^+^ exchanger 6 (*NHE6*) gene have been linked to epilepsy, speech loss, truncal ataxia, hyperkinesia, and postnatal microcephaly.

**Methods:**

In the present study, we evaluated genetic alterations in a 3‐year‐old Chinese boy displayed features of epilepsy, psychomotor retardation, microcephaly, low body weight, difficulty in feeding, excessive movement, attention loss, ataxia, and cerebellar atrophy and his healthy family using WES method. The identified variant was further confirmed by Sanger sequencing method. Finally, minigene assays were used to verify whether the novel *SLC9A6* intronic variant influenced the normal splicing of mRNA.

**Results:**

We identified a novel hemizygous splicing variant [NM_001042537.1: c.1463‐1G>A] in *SLC9A6* by trio‐based exome sequencing. The minigene expression in vitro confirmed the splicing variant altered a consensus splice acceptor site of *SLC9A6* intron 11, resulting in skipping over exon 12.

**Conclusions:**

Our finding extends the catalog of pathogenic intronic variants affecting *SLC9A6* pre‐mRNA splicing and provides a basis for the genetic diagnosis of CS.

## Graphical abstract

We report on a case of 3‐year‐old Chinese boy with Christianson Syndrome (CS) displayed the features of epilepsy, psychomotor retardation, microcephaly, low body weight, difficulty in feeding, excessive movement, attention loss, ataxia, and cerebellar atrophy. We identified a novel homozygous splicing variant [c.1463–1 (IVS11) G >A] in *SLC9A6* by trio‐based exome sequencing. The minigene expression in vitro confirmed the splicing variant altered a consensus splice acceptor site of *SLC9A6* intron 11, resulting in skipping over exon 12. Our finding extends the catalog of pathogenic intronic mutations affecting *SLC9A6* pre‐mRNA splicing and provides a basis for the genetic diagnosis of CS.

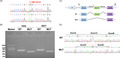

## 1 INTRODUCTION

Solute carrier family 9 member A6 gene (*SLC9A6*, OMIM 300231) is located at Xq26.3 and encodes the alkali cation (Na^+^,K^+^)/proton (H^+^) exchanger NHE6 isoform protein, which plays a critical role in the regulation of pH lumen in early recycling endosomes and the process of neuronal arborization and synapse development.^1^, ^2^, ^3^ The alteration of *SLC9A6* causes a phenotype mimicking Angelman Syndrome, now referred to as Christianson syndrome (CS,OMIM 300243).^4^ To date, at least 35 different *SLC9A6* pathogenic variants have been identified.

CS is an X‐linked neurodevelopmental and neurological disorder, characterized in males by critical symptoms that include intellectual disability, epilepsy, hyperkinesis, truncal ataxia, and postnatal‐onset microcephaly.^1^, ^5^, ^6^ Furthermore, these core phenotypic features can also be accompanied by secondary symptoms, such as autistic behaviors, poor weight gain, feeding difficulties, motor regression, sleep disturbances, squint, high pain threshold, hypotonia, gastroesophageal reflux, and different degrees of cerebellar atrophy (CA).^7^, ^8^, ^9^ Contrast to male, female variant carriers show milder phenotypes with variable penetrance.^4^, ^10^ Affected CS patients show a remarkable neurological symptom may be due to high‐level expression of mutant *SLC9A6* gene in the central nervous system.^11^, ^12^

To date, few Chinese CS patients have been reported. Here, we report a Chinese boy presenting with Christianson syndrome due to novel splicing variant NM_001042537.1: c.1463‐1G>A in the *SLC9A6* gene. The variant is not mentioned in ClinVar, dbSNP, 1000genomes, NCBI, ExAC, or HGMD database. We performed minigene splicing assay to validate the functional effect of variant in *SLC9A6* gene to determine whether the hemizygous site is a pathogenic site for the affected boy.

## 2 MATERIALS AND METHODS

### 2.1 Patient and ethics

The present study was approved by the Ethics Committee of Northwest women's and children's hospital (Xi'an, China). Written informed consent was obtained from the parents.

### 2.2 Samples and DNA extraction

Genomic DNA was extracted from 2–3 ml EDTA peripheral blood sample using BloodGen Midi Kit (CWBIO), according to the manufacturer's protocol.

### 2.3 Variant screening

Library enrichment for whole exome sequencing was conducted using SeqCap EZ Exome v3 kit (Roche NimbleGen). Then, the enriched samples were sequenced by using an Illumina Hiseq 2500 (Illumina). The variants which is presented at least 20% of all reads were identified as quality variants. Thus, all quality variants were evaluated by bioinformatic tools including Polyphen2, PROVEAN, and MaxEntScan. The pathogenicity of all quality variants was accessed by The American College of Medical Genetics and Genomics (ACMG) Standards and Guidelines.^13^ The pathogenic variant was validated by Sanger sequencing.

### 2.4 Transcription analysis

*SLC9A6* fragments containing intron11, exon12, and intron12 were amplified from DNA of patient and control by nested PCR. The first PCR was performed using genomic DNA as a template, with 37527‐SLC9A6‐F and 39956‐SLC9A6‐R as primers; the second PCR was performed using products from the first PCR as a template, with 37833‐SLC9A6‐F and 39651‐SLC9A6‐R as primers; the third PCR was performed using PCR products from the second round as a template, with pcMINI‐SLC9A6‐KpnI‐F and pcMINI‐SLC9A6‐BamHI‐R as primers. The primer sequences were listed as Table 1.

**TABLE 1:** Primers for transcription analysis

Primers	Sequence (5’−3’)
37527‐SLC9A6‐F	ggcatgcaccaatatgcctg
37833‐SLC9A6‐F	ctgattccaaatccttggtt
39651‐SLC9A6‐R	ttttatctgacctggtgata
39956‐SLC9A6‐R	gtattctggaagcttggtag
pcMINI‐SLC9A6‐KpnI‐F	ggtaggtaccctgggattacaggcatgcgc
pcMINI‐SLC9A6‐BamHI‐R	tagtggatccgtaaatgtccatgctatatt

Restriction enzymes Kpn I and BamH I were used to digest amplified products, and the digestion products were ligated to pcMINI plasmid to obtain the pcMINI‐SLC9A6‐wt and pcMINI‐SLC9A6‐mut plasmid (Figure 4A). The recombinant plasmids were then transfected into human embryonic kidney cells (HEK‐293T) and human cervical cancer cells (HeLa) by lipofectamine 2000 (Invitrogen). After 48 h, cells were harvested and RNA was extracted by RNAiso Plus (TAKARA). cDNA was synthesized using PrimeScript RT Master Mix (TAKARA). Finally, the cDNA was identified using 1.8% agarose gel electrophoresis and verified through sequencing.

## 3 RESULT

### 3.1 Clinical case report

The boy was born in full‐term normal delivery without anoxia and asphyxia history. His birth weight, length, and occipitofrontal circumference (OFC) were reported to be within normal range. There were no family histories of mental illness, genetic illness, epilepsy, and febrile seizures. His six‐month‐old sister was in good health. His parents were both Han Chinese.

He started a seizure at the age of 12 months, characterized by general tonic‐clonic seizure, and has been treated with topiramate (TPM) firstly. TPM was stopped using without permission from doctor after half a month. However, 3 months later, he had frequent general tonic‐clonic seizures in clusters for 6 months, despite antiepileptic treatment by sodium valprorate (VPA). General tonic‐clonic seizures were suddenly completely controlled by additional use of levoethylacetam (LEV) and repetitive transcranial magnetic stimulation (rTMS), with a seizure‐free interval between 22 months old and 34 months old. During the absence of seizures, rTMS has been added only a few times and then discontinued, LEV was replaced with lamotrigine (LTG) because of his hyperkinesis and bad appetite after using 6 months, and LTG was subsequently replaced with zonisamide (ZNS) because of his anaphylaxis after using one month. But nocturnal seizures were found at age of 34 months, characterized by consciousness loss and upward gaze, without other abnormal movements. Oxcarbazepine (OXZ) was added to the antiepileptic treatment in external hospital. However, general tonic‐clonic seizures in clusters were appeared again at the age of 39 months, subsequently followed by myoclonic and atonic seizure in clusters. Just then, he was admitted to our hospital.

He was initially diagnosed as psychomotor retardation at the age of 9 months, due to a visible delaying of developmental milestones, including unstable sitting posture and undeveloped language. The first electroencephalo graph (EEG) and magnetic resonance imaging (MRI) indicated nothing abnormal. And he received rehabilitation training regularly and achieved independent ambulation at the age of 2 years and 6 months finally, but his gait was insecure and ataxic. He had no autistic behaviors so far, but had hyperactive and distracted behaviors. He spoke little, occasionally, some unconscious reduplicated words. He had poor weight gain because of having poor appetite and frequent gastrointestinal discomfort. He hardly ever drooled.

On physical examination, his weight was 11 kg (<1st centile), belonged to severe underweight, his OFC 45 cm (<1st centile), belonged to microcephaly. His mandibular tension was high and with facial obesity, often closed teeth, restricted opening mouth. The neurological examination was unremarkable except the ataxic gait.

During hospitalization, most of the laboratory test results were normal, which included ammonia, homocysteine, inorganic phosphorus, serum calcium, 25‐(OH)D, ceruloplasmin, thyroid function, erythrocyte sedimentation rate (ESR), urine organic acids analysis, and karyotype. Thus, only serum free carnitine concentration slightly reduced (7.68 μmol/L, normal range: 8.5–50 μmol/L).

**FIGURE 1:**
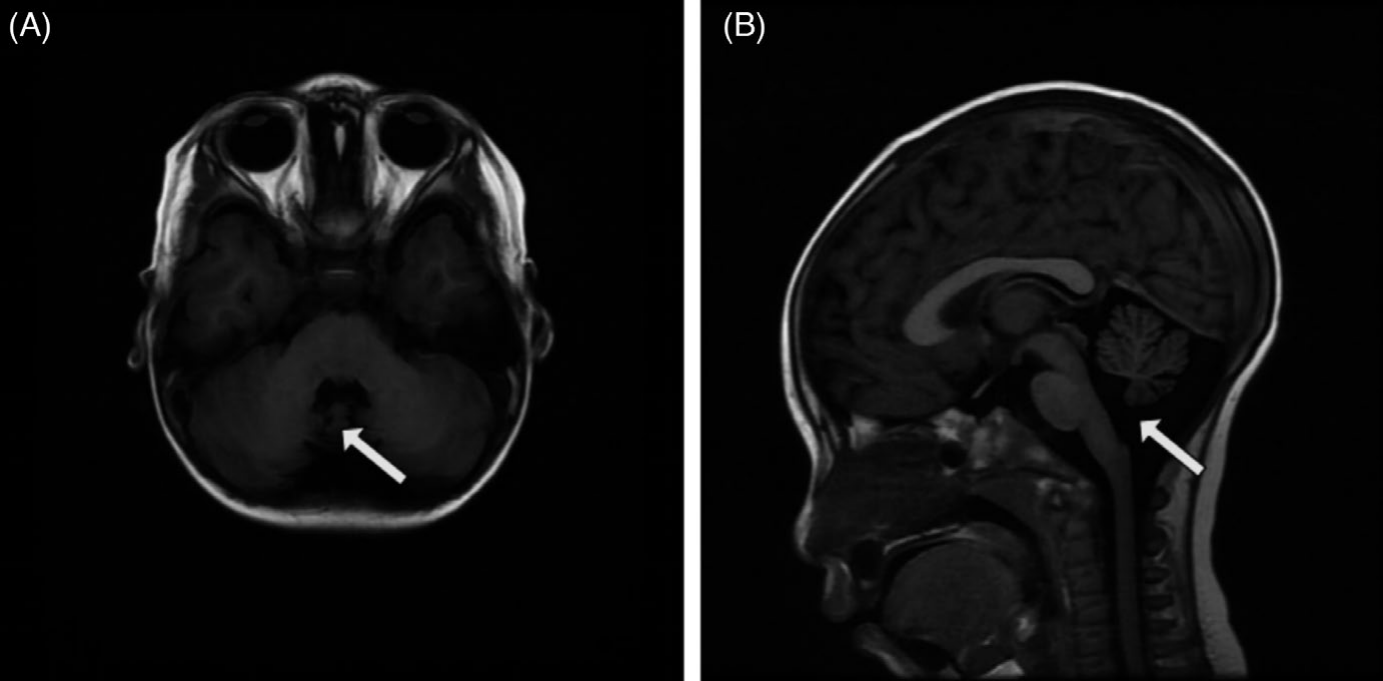
Brain MRI findings. (A) Axial T1‐weighted image showing dysplasia of inferior cerebellar vermis. (B) Mid‐sagittal T1‐weighted image showing cerebellar atrophy mainly in the vermis, and the fourth ventricle enlarged

**FIGURE 2:**
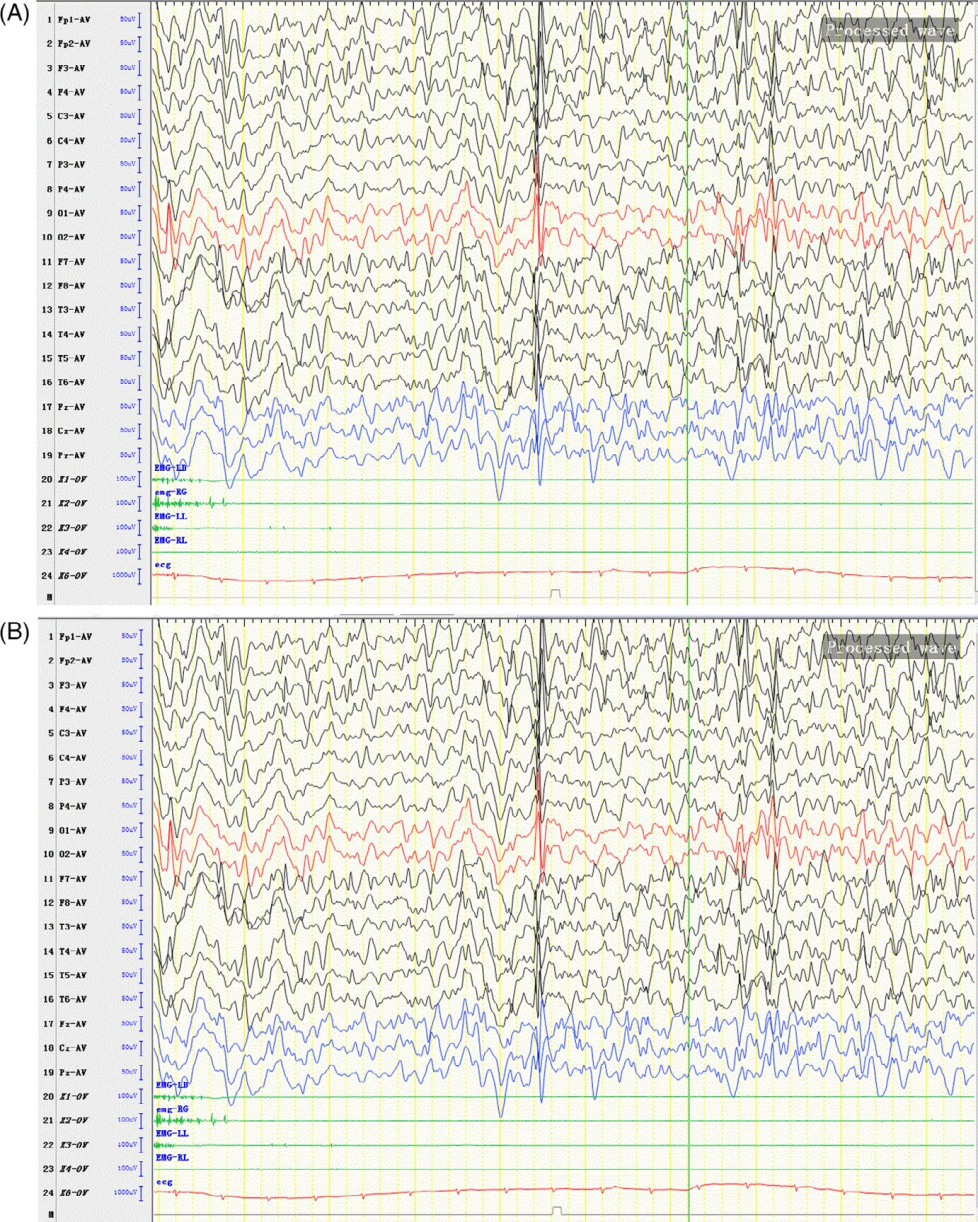
EEG findings. (A) Awake EEG revealing on the basis of diffuse irregular medium to high amplitude 4–6 Hzθ rhythm, middle to very high amplitude spikes, multiple spikes, spikes and slow waves complex, and rhythms were exploded frequently and asynchronously. The leads of frontal, occipital, and temporal were prominent. The right hemisphere was more obvious than the left. (B) Sleep EEG revealing frequent unsynchronized paroxysm of middle to very high amplitude spikes, mutiple spikes, spikes and slow waves complex, and rhythms, over bilateral anterior‐middle frontal, occipital, and temporal. The right hemisphere was more obvious. Epileptiform discharge was significantly increased than being awake

Furthermore, EEG and MRI were rechecked. Awake EEG revealed that on the basis of diffuse irregular medium to high amplitude 4–6 Hz θ rhythm, middle to very high amplitude spikes, multiple spikes, spikes and slow waves complex, and rhythms were exploded frequently and asynchronously. The leads of frontal, occipital, and temporal were prominent. The right hemisphere was more obvious than the left. During sleep, epileptiform discharge was significantly increased than awake (see Figure 1). The brain MRI revealed hypoplasia of inferior parts of cerebellar vermis, and enlargement of inferior orifice of the fourth ventricle, and abnormal signals in the left frontal cortex. The other intracranial structures including brainstem, basal ganglia, and supratentorial brain structures were within normal limits (Figure 2).

Since he came out of our hospital, there has been a seizure‐free interval of 5 months. During this period, his ambulation and appetite were a little better than before, but his language development and weight gain still changed little. He still had no autistic behavior, but he was easily irritated. He was more likely to suffer from upper respiratory infections than normal children, sometimes even had fever, but there was no seizure. General tonic‐clonic, myoclonic, and atonic seizures were no longer appeared. But sleep seizures occurred occasionally again after 5 months, characterized by consciousness loss and upward gaze, without other abnormal movements, and relieved after about 3 minutes. The dose of LEV was adjusted from 30 to 60 mg/kg every day. After each dose adjustment, the frequency of seizures would be significantly reduced, but sleep seizures were not controlled completely.

### 3.2 The whole exome sequencing reveals a novel *SLC9A6* splicing variant

**FIGURE 3:**
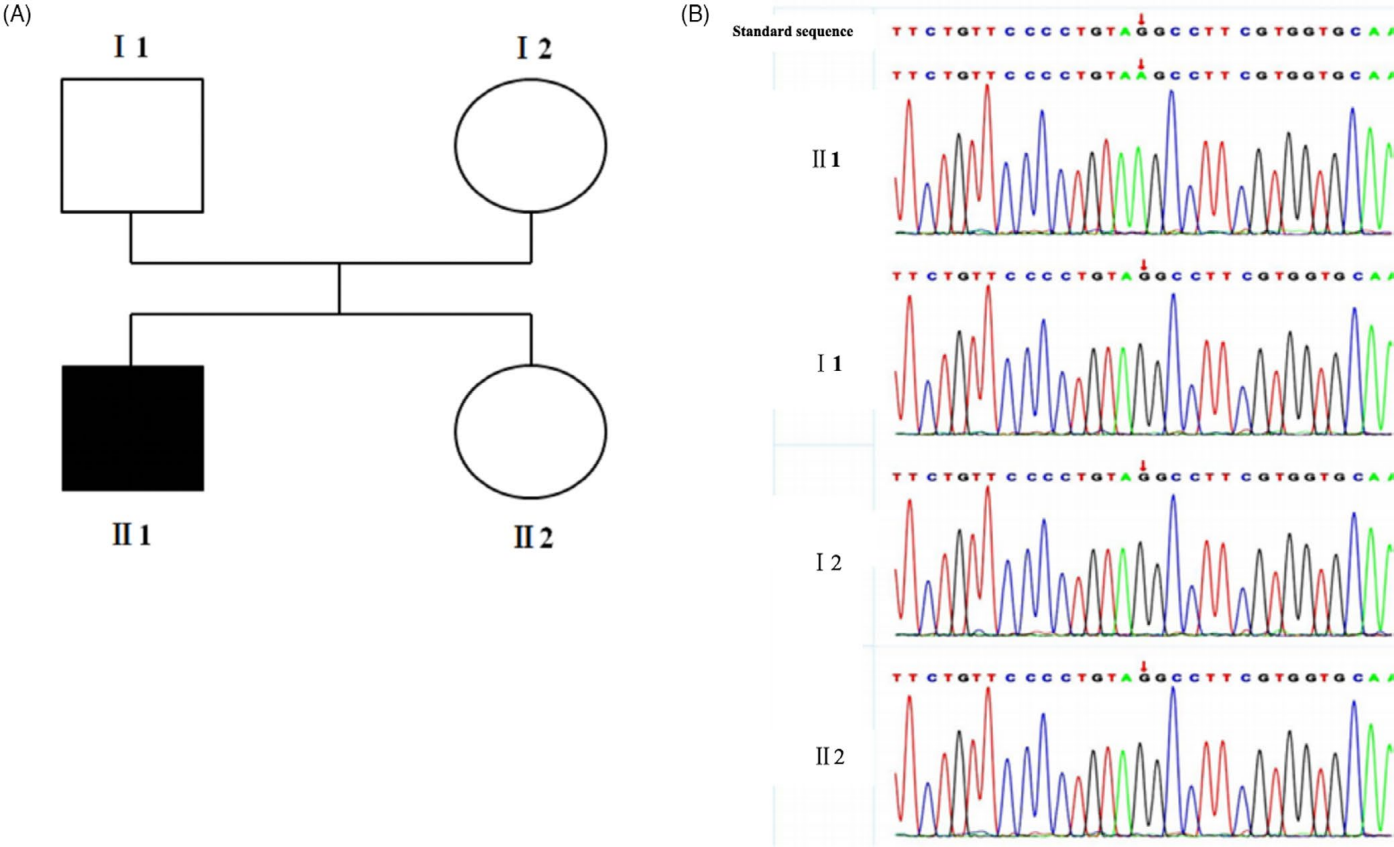
A. Family's pedigree and variant. Pedigree of the family. B. Sanger sequencing revealed a splice‐site variant in *SLC9A6* gene

Whole exome sequencing was performed on the proband and his parents and sister, and variant filtering was performed as described in the methods. Annotated whole exome sequencing data were examined for variants in genes for relevance for the epilepsy and developmental delay phenotypes. Finally, one splicing variant was chosen as potential causal variant. The splicing variant NM_001042537.1: c.1463‐1G>A was not previously reported in variant databases including ClinVar, dbSNP, 1000genomes, NCBI, ExAC, and HGMD database. Sanger sequencing confirmed the hemizygous variant of the *SLC9A6* in the proband II‐1, which was a *de novo* variant not founded in his parents and sister (Figure 3).

### 3.3 The intron c.1463‐1G >A variant causes a skipping transcription of exon 12

**FIGURE 4:**
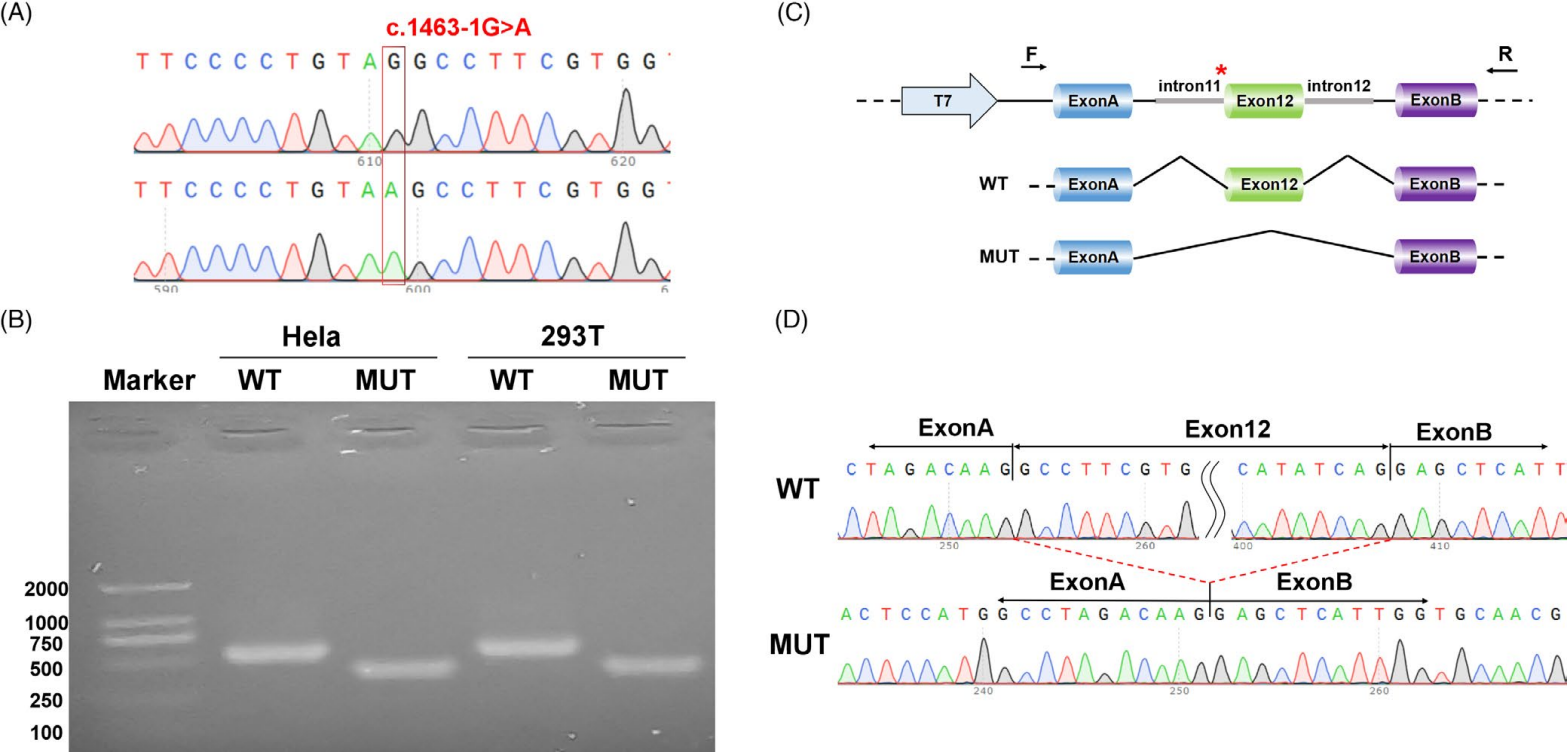
Splicing analysis using a minigene assay. (A) Construction of the pcMINI‐SLC9A6‐wt and pcMINI‐SLC9A6‐mut plasmid. (B) Reverse‐transcription polymerase chain reaction (RT‐PCR) products were separated by electrophoresis of the pcMINI‐SLC26A4‐wt/mut vector in HeLa and 293T cells. The longer band represented the wild‐type transcript (WT) with a length of 543 bp, the shorter band represented the mutated transcript (MT) with a length of 249 bp. (C) Schematic diagram of minigene construction and schematic diagram of Sanger sequencing of RT‐PCR products. (D) Sanger sequencing of the RT‐PCR products, the wild‐type transcript consisted of exon A, 12, and B, in contrast, the mutated transcript consisted of exon A and B, without exon 12

In vitro transcription analysis was performed to validate how the variant affect splicing products. Figure 4B showed that mutant plasmid encoded a shorter transcriptional products compared to the WT plasmid. The results of Sanger sequencing demonstrated the intron mutant lead to a complete skipping of exon 12 (Figure 4C, Figure 4D).

## 4 DISCUSSION

According to our literature review and this study, at least 35 different *SLC9A6* pathogenic variants have been identified, which included 16 exonic variants and 10 intronic splices site variants (Table 2).^3^, ^5^, ^6^, ^9^, ^14^, ^15^, ^16^, ^17^, ^18^, ^19^, ^20^, ^21^, ^22^, ^23^, ^24^, ^25^, ^26^, ^27^, ^28^, ^29^, ^30^ The exonic mutational spectrum showed that exon 12 was a high incidence region of pathogenic variants, accounting for 43.8% of all exonic pathogenic variants. SLC9A6 is a twelve‐span transmembrane protein and exon 12 encodes the last transmembrane motif.^3^ The mutational spectrum indicated a vital role of exon 12 in the function of SLC9A6. The *de novo* splice‐site variant [NM_001042537.1: c.1463‐1G>A] in *SLC9A6* was identified as a novel pathogenic variant in this report. This variant is closest variant to C‐terminal of the known pathogenic intron variant, and it causes the skipping transcription of exon 12. Given the importance of exon 12, the pathogenicity of this variant is predicated.

**TABLE 2:** Summary of the SLC9A6 variants of CS patients

Alteration region	Alteration type	Variants (default transcript NM_001042537)	Protein alteration	Clinical feature	References
Exon 1	Nonsense variant	NM_006359: c.190G>T	p. Glu64*	Mental retardation, epilepsy, ataxia, drooling	Pescosolido et al., 2014
Exon 1	Missense variant	c.316A>G	p. Met106Val	Mild mental retardation, severe behavioral disturbances	Ibarluzea et al. 2020
Exon 2	Frame shift variant	c.441delG	p. Ser147fs	Microcephaly, dysphasia, epilepsy, ataxia, strabismus, drooling, no cerebellar atrophy	Takahashi et al.2011
Exon 3	Frame shift variant	NM_006359: c.477_481del	p. Ile160Leufs*5	Severe mental retardation, generalized tonic seizure, microcephaly, cerebellar atrophy, ataxia, hypotonia	Ikeda et al.2019
Exon 3	Frame shift variant	c.582_595del	p. Tyr194fs	Focal seizures (impaired awareness), no cerebellar atrophy	Liu et al.2018
Exon 4	Frame shift variant	NM_006359: c.512_513delAT	p. His171fs	Mental retardation, microcephaly, epilepsy, ophthalmoplegia, squint, motor regression, ataxia	Christianson et al., 1999
Exon 4	Frame shift variant	c.540_547dupAGAAGTAT	p. Phe183fs*1	Mental retardation, epilepsy, microcephaly, ataxia, drooling, autism, hypotonia, no cerebellar atrophy	Pescosolido et al., 2014
Exon 5	Frame shift variant	NM_006359: c.608del	p. His203Leufs*10	Developmental delay, seizure, movement disorder, dystonia	Trump et al. 2016
Exon 6	Non‐frame shift variant	NM_006359: c.764_769delAAAGTG	p. Glu255_Ser256del	Developmental delay, epilepsy,ataxia,microcephaly, verbal language absent, easily provoked laughter	Gilfillan et al., 2008
Exon 6	Frame shift variant	c.838_839delinsG	p. Leu280Alafs*17	Severe mental retardation, microcephaly, generalized seizure, autism	Fung et al.2017
Exon 7	Non‐frame shift variant	c.1012_1020del	p. Trp338_Thr340del	Severe mental retardation, dysphasia, autism, ataxia, motor regression, unilateral weakness, no cerebellar atrophy	Garbern et al., 2010
Exon 7	Nonsense variant	c.916C>T	p. Gln306*	Mental retardation, microcephaly, cerebellar atrophy, epilepsy, ophthalmoplegia, squint, drooling, flexed arms, motor regression, ataxia, dystonia	Mignot et al., 2013
Exon 9	Nonsense variant	c.1219C>T	p. Gln407*	Mental retardation, epilepsy, microcephaly, ataxia, drooling, autism, hypotonia, cerebellar atrophy	Schroer et al., 2010
Exon 9	Frame shift variant	NM_006359: c.1222_1226del	p. His408Asnfs*2	early infantile epileptic encephalopathy, dystonia	Trump et al. 2016
Exon 10	Splice‐site variant and missense variant	c.1148G>A	p. Gly383Asp	Mental retardation, epilepsy, microcephaly, ataxia, drooling, autism, no cerebellar atrophy	Pescosolido et al., 2014
Exon 11	Frame shift variant	c.1414dupA	p. Arg472fs*4	Mental retardation, epilepsy, microcephaly, ataxia, drooling, autism, hypotonia, cerebellar atrophy	Pescosolido et al., 2014
Exon 12	Nonsense variant	c.1498C>T	p. Arg468*	Developmental delay, epilepsy,ataxia,microcephaly, verbal language absent, easily provoked laughter, no cerebellar atrophy	Gilfillan et al., 2008
Exon 12	Frame shift variant	NM_006359: c.1464_1465insT	p. Thr489Tyrfs*23	Severe mental retardation, microcephaly, seizure, scoliosis	Riess et al., 2012
Exon 12	Frame shift variant	c.1505_1509dupCTGCC	p. Thr504Leufs*8	Mental retardation, suspicion of epilepsy, microcephaly	Yalcintepe et al. 2021
Exon 12	Nonsense variant	c.1568G>A	P. Trp523*	Mental retardation, epilepsy, microcephaly, ataxia, drooling, autism, hypotonia, no cerebellar atrophy	Pescosolido et al., 2014
Exon 12	Nonsense variant	c.1569G>A	p. Trp523*	Mental retardation, epilepsy, electrical status epilepticus in sleep, autism	Mathieu et al., 2018
Exon 13	Nonsense variant	c.1639G>T	p. Glu547*	Mental retardation, epilepsy, microcephaly, ataxia, happy behavior	Schuurs‐Hoeijmakers et al., 2013
Exon 14	Nonsense variant	c.1710G>A	p. Trp570*	Mental retardation, epilepsy, microcephaly, ataxia, drooling, autism, hypotonia,	Pescosolido et al., 2014
Exon 15	Missense variant	NM_006359: c.1831G>A	p. Glu611Lys	Global developmental delay, cerebellar atrophy, focal and generalized tonic‐clonic seizures, hypotonia, large head	Padmanabha et al.2017
Intron 2	Splice‐site variant	c.526–1,G>A	‐	Mental retardation, epilepsy, microcephaly, ataxia, drooling, autism, hypotonia, cerebellar atrophy	Pescosolido et al., 2014
Intron 2	Splice‐site variant	c.526‐9_526‐5del	Skipping of exon 3 (mRNA validation)	Mild Mental retardation, Dysphasia, No autistic behavior	Masurel‐Paulet A et al. 2016
Intron 3	Splice‐site variant	NM_006359: c.507+1delGTAA	p.V144_R169 del	Developmental delay, epilepsy,ataxia,microcephaly, verbal language absent, easily provoked laughter, no cerebellar atrophy	Gilfillan et al., 2008
Intron 4	Splice‐site variant	NM_006359: c.584+1G>T	‐	Severe mental retardation, microcephaly, seizure, strabismus	Riess et al., 2012
Intron 4	Splice‐site variant	NM_006359: c.584+5G>A	‐	Global developmental delay, febrile seizure, delayed myelination, ataxia	Mercimek‐Mahmutoglu S, et al. 2015
Intron 5	Splice‐site variant	c.794‐2A>G	‐	Severe mental retardation, microcephaly, focal seizure, autism	Fung et al.2017
Intron 6	Splice‐site variant	NM_006359: c.899+3_899+6del	Skipping of exon 6 (mRNA validation)	Mental retardation, microcephaly, hearing impairment, MRI was normal	Zhang et al. 2020
Intron 9	Splice‐site variant	NM_006359: c.1141‐8C>A	multiple aberrant transcripts (mRNA validation)	Severe developmental delay, microcephaly, epilepsy, ataxia,verbal language absent, MRI was normal	Ieda et al.2019
Intron 10	Splice‐site variant	c.1151‐1G>A	‐	Mental retardation, microcephaly, electrical status epilepticus in sleep, ataxia, cerebellar atrophy,	Zanni et al., 2014
Intron 11	Splice‐site variant	c.1463‐1G>A	Skipping of exon 12 (mRNA validation)	Mental retardation, epilepsy, microcephaly, verbal language absent, ataxia, cerebellar atrophy	This report

This patient presented drug refractory epilepsy, global psychomotor development delay, microcephaly, underweight, feeding difficulties, hyperkinesis, attention‐deficit, ataxic gait and mild CA, no mouth opening, and no drooling and had a de novo splice‐site variant in *SLC9A6* on the X chromosome which caused a skipping transcription of exon 12. Consequently, the patient was diagnosed as CS definitely.

The clinical presentations of this patient were almost consistent with the characteristics of CS, except for no mouth opening, no drooling, and no autistic behavior; many previous literatures also have described the above symptoms as secondary symptom.^3^, ^31^ The detailed mechanism might be related to different variants of *SLC9A6* gene leading to different phenotypes of CS.

According to the previous reports, CS specific brain MRI presented as CA. CA occurred mainly in the inferior parts of vermis and cerebellar hemisphere, especially in the cerebellar vermis.^7^ This patient has received two brain MRI scans at the age of 9 month and 3 year, respectively. The first brain MRI showed normal intracranial structures, but the later brain MRI showed the inferior parts of cerebellar vermis dysplasia and the inferior orifice of the fourth ventricle enlargement (Figure 1), which indicated that the cerebellum was slowly atrophy and its further development should be monitored during follow‐up. It was reported that the occurrence of CA was mainly related to the extensive progressive loss and degeneration of cerebellar Purkinje cells caused by *SLC9A6* gene variant in the CS.^21^ The above results indicated that the occurrence of CA was progressive in the CS. According to the neuroimaging data of 17 CS patients, the average age of onset of CA was about 11 years old.^7^ Therefore, although the brain MRI scans indicate no abnormality, the possibility of CS should also be considered in the patients with mental retardation less than 1 year of age. Their brain MRI should be monitored constantly and regularly after onset.

There are still some limitation in this study. On the one hand, transcription analysis in the patient was not investigated. It was reported that peripheral blood mRNA analysis is helpful for ascertainment of alternative splicing of *SLC9A6* induced CS.^29^ On the other side, the correlation between genotype and phenotype is still need further investigation.

In conclusion, few Chinese CS patients have been reported based on the current literature. This case described the clinical manifestations of a Chinese CS patient, including epilepsy, microcephaly, ataxia, and progressive cerebellar atrophy. *SLC9A6* NM_001042537.1: c.1463‐1G>A was found to be a pathogenic variant. The novel finding broadens the spectrum of *SLC9A6* gene variants in CS and provides a basis for the genetic diagnosis of CS.

## CONFLICT OF INTEREST

The authors declare that they have no competing interests.

## Data Availability

The data used to support the findings of this study are included within the article. And the raw data used to support the findings of this study are available from the corresponding author upon request.

## References

[1] Fichou Y , Bahi‐Buisson N , Nectoux J , et al. Mutation in the *SLC9A6* gene is not a frequent cause of sporadic Angelman‐like syndrome. Eur J Hum Genet. 2009;17(11):1378‐1380. 19471312 10.1038/ejhg.2009.82 PMC2986687

[2] Ouyang Q , Lizarraga SB , Schmidt M , et al. Christianson syndrome protein NHE6 modulates TrkB endosomal signaling required for neuronal circuit development. Neuron. 2013;80(1):97‐112. 24035762 10.1016/j.neuron.2013.07.043 PMC3830955

[3] Pescosolido MF , Stein DM , Schmidt M , et al. Genetic and phenotypic diversity of NHE6 mutations in Christianson syndrome. Ann Neurol. 2014;76(4):581‐593. 25044251 10.1002/ana.24225 PMC4304796

[4] Sinajon P , Verbaan D , So J . The expanding phenotypic spectrum of female *SLC9A6* mutation carriers: a case series and review of the literature. Hum Genet. 2016;135(8):841‐850. 27142213 10.1007/s00439-016-1675-5

[5] Garbern JY , Neumann M , Trojanowski JQ , et al. A mutation affecting the sodium/proton exchanger, SLC9A6, causes mental retardation with tau deposition. Brain. 2010;133(Pt 5):1391‐1402. 20395263 10.1093/brain/awq071 PMC2859154

[6] Mignot C , Héron D , Bursztyn J , et al. Novel mutation in *SLC9A6* gene in a patient with Christianson syndrome and retinitis pigmentosum. Brain Dev. 2013;35(2):172‐176. 22541666 10.1016/j.braindev.2012.03.010

[7] Bosemani T , Zanni G , Hartman AL , et al. Christianson syndrome: spectrum of neuroimaging findings. Neuropediatrics. 2014;45(4):247‐251. 24285247 10.1055/s-0033-1363091

[8] Ilie A , Gao AY , Reid J , et al. A Christianson syndrome‐linked deletion mutation (∆(287)ES(288)) in *SLC9A6* disrupts recycling endosomal function and elicits neurodegeneration and cell death. Mol Neurodegener. 2016;11(1):63. 27590723 10.1186/s13024-016-0129-9 PMC5010692

[9] Takahashi Y , Hosoki K , Matsushita M , et al. A loss‐of‐function mutation in the *SLC9A6* gene causes X‐linked mental retardation resembling Angelman syndrome. Am J Med Genet B Neuropsychiatr Genet. 2011;156B(7):799‐807. 21812100 10.1002/ajmg.b.31221

[10] Sinajon P , Verbaan D , So J . The expanding phenotypic spectrum of female *SLC9A6* mutation carriers: a case series and review of the literature. Hum Genet. 2016;135(8):841‐850. 27142213 10.1007/s00439-016-1675-5

[11] Pescosolido MF , Kavanaugh BC , Pochet N , et al. Complex neurological phenotype in female carriers of NHE6 mutations. Mol Neuropsychiatry. 2019;5(2):98‐108. 31192222 10.1159/000496341 PMC6528080

[12] Fagerberg L , Hallström BM , Oksvold P , et al. Analysis of the human tissue‐specific expression by genome‐wide integration of transcriptomics and antibody‐based proteomics. Mol Cell Proteomics. 2014;13(2):397‐406. 24309898 10.1074/mcp.M113.035600 PMC3916642

[13] Richards S , Aziz N , Bale S , et al. Standards and guidelines for the interpretation of sequence variants: a joint consensus recommendation of the American college of medical genetics and genomics and the association for molecular pathology. Genet Med. 2015;17(5):405‐424. 25741868 10.1038/gim.2015.30 PMC4544753

[14] Ibarluzea N , Hoz AB , Villate O , et al. Targeted next‐generation sequencing in patients with suggestive X‐linked intellectual disability. Genes (Basel). 2020;11(1):51. 10.3390/genes11010051 PMC7017351 31906484

[15] Ieda D , Hori I , Nakamura Y , et al. A novel splicing mutation in *SLC9A6* in a boy with Christianson syndrome. Hum Genome Var. 2019;6:15. 30937176 10.1038/s41439-019-0046-x PMC6434044

[16] Liu J , Tong L , Song S , Niu Y , Li J , Wu X , et al. Novel and de novo mutations in pediatric refractory epilepsy. Mol Brain. 2018;11(1):48. Erratum in: Mol Brain. 2018;11(1):59. 30185235 10.1186/s13041-018-0392-5 PMC6125990

[17] Christianson AL , Stevenson RE , van der Meyden CH , et al. X linked severe mental retardation, craniofacial dysmorphology, epilepsy, ophthalmoplegia, and cerebellar atrophy in a large South African kindred is localised to Xq24‐q27. J Med Genet. 1999;36(10):759‐766. 10528855 10.1136/jmg.36.10.759 PMC1734236

[18] Trump N , McTague A , Brittain H , et al. Improving diagnosis and broadening the phenotypes in early‐onset seizure and severe developmental delay disorders through gene panel analysis. J Med Genet. 2016;53(5):310‐317. 26993267 10.1136/jmedgenet-2015-103263 PMC4862068

[19] Gilfillan GD , Selmer KK , Roxrud I , et al. *SLC9A6* mutations cause X‐linked mental retardation, microcephaly, epilepsy, and ataxia, a phenotype mimicking Angelman syndrome. Am J Hum Genet. 2008;82(4):1003‐1010. 18342287 10.1016/j.ajhg.2008.01.013 PMC2427207

[20] Fung CW , Kwong AK , Wong VC . Gene panel analysis for nonsyndromic cryptogenic neonatal/infantile epileptic encephalopathy. Epilepsia Open. 2017;2(2):236‐243. 29588952 10.1002/epi4.12055 PMC5719849

[21] Schroer RJ , Holden KR , Tarpey PS , et al. Natural history of Christianson syndrome. Am J Med Genet A. 2010;152A(11):2775‐2783. 20949524 10.1002/ajmg.a.33093 PMC3698558

[22] Riess A , Rossier E , Krüger R , et al. Novel *SLC9A6* mutations in two families with Christianson syndrome. Clin Genet. 2013;83(6):596‐597. 22931061 10.1111/j.1399-0004.2012.01948.x

[23] Yalcintepe S , Gurkan H . Novel c.1505_1509dupCTGCC pathogenic variation in a male case with Christianson syndrome. Clin Dysmorphol. 2021;30(1):36‐38. 33278113 10.1097/MCD.0000000000000358

[24] Mathieu ML , de Bellescize J , Till M , et al. Electrical status epilepticus in sleep, a constitutive feature of Christianson syndrome? Eur J Paediatr Neurol. 2018;22(6):1124‐1132. 30126759 10.1016/j.ejpn.2018.07.004

[25] Schuurs‐Hoeijmakers JH , Vulto‐van Silfhout AT , Vissers LE , et al. Identification of pathogenic gene variants in small families with intellectually disabled siblings by exome sequencing. J Med Genet. 2013;50(12):802‐811. 24123876 10.1136/jmedgenet-2013-101644

[26] Padmanabha H , Saini AG , Sahu JK , Singhi P . Syndrome of X linked intellectual disability, epilepsy, progressive brain atrophy and large head associated with *SLC9A6* mutation. BMJ Case Rep. 2017;2017:bcr2017222050. 10.1136/bcr-2017-222050 PMC5780601 29275387

[27] Masurel‐Paulet A , Piton A , Chancenotte S , et al. A new family with an *SLC9A6* mutation expanding the phenotypic spectrum of Christianson syndrome. Am J Med Genet A. 2016;170(8):2103‐2110. 27256868 10.1002/ajmg.a.37765

[28] Mercimek‐Mahmutoglu S , Patel J , Cordeiro D , et al. Diagnostic yield of genetic testing in epileptic encephalopathy in childhood. Epilepsia. 2015;56(5):707‐716. 25818041 10.1111/epi.12954

[29] Zhang X , Qiu W , Liu H , et al. RT‐PCR analysis of mRNA revealed the splice‐altering effect of rare intronic variants in monogenic disorders. Ann Hum Genet. 2020;84(6):456‐462. 32776513 10.1111/ahg.12400

[30] Zanni G , Barresi S , Cohen R , et al. A novel mutation in the endosomal Na^+^/H^+^ exchanger NHE6 (*SLC9A6*) causes Christianson syndrome with electrical status epilepticus during slow‐wave sleep (ESES). Epilepsy Res. 2014;108(4):811‐815. 24630051 10.1016/j.eplepsyres.2014.02.009

[31] Deane EC , Ilie AE , Sizdahkhani S , Das Gupta M , Orlowski J , McKinney RA . Enhanced recruitment of endosomal Na^+^/H^+^ exchanger NHE6 into Dendritic spines of hippocampal pyramidal neurons during NMDA receptor‐dependent long‐term potentiation. J Neurosci. 2013;33(2):595‐610. 23303939 10.1523/JNEUROSCI.2583-12.2013 PMC6704919

